# The diagnostic value of dual-energy CTA for visualising below the knee arteries in peripheral arterial disease: A systematic review

**DOI:** 10.1016/j.ejro.2025.100704

**Published:** 2025-11-07

**Authors:** Jade Baars, Thijs Urlings, Edwin van der Linden, Ilia Panfilov, Lodewijk Cobben, Willem-Jan de Jong, Joost van der Vorst, Jaap Hamming, Ayoub Charehbili

**Affiliations:** a Department of Radiology, Haaglanden Medical Center, Lijnbaan 32, The Hague 2512 HH, the Netherlands; b Department of Vascular Surgery, Leiden University Medical Center, Albinusdreef 2, Leiden 2333 ZA, the Netherlands; c Department of Vascular Surgery, Haaglanden Medical Center, Lijnbaan 32, The Hague 2512 HH, the Netherlands

**Keywords:** Peripheral arterial disease, Dual energy, CT angiography, Virtual monochromatic, Imaging, Lower legs

## Abstract

**Background:**

Computed tomography angiography (CTA) of below the knee (BTK) arteries in patients with peripheral arterial disease (PAD) is often challenging due to reduced contrast enhancement and the presence of arterial wall calcifications. Several studies have been published using dual energy (DE) techniques to improve image quality and diagnostic performance of CTA. This systematic review aims to assess diagnostic performance of dual energy CTA (DECTA) in BTK arteries of patients with PAD.

**Methods:**

A systematic literature search was conducted to identify studies reporting on DECTA of BTK arteries. Studies were included if they assessed imaging quality using qualitative or objective parameters, or provided data on diagnostic accuracy. The search was performed in Pubmed, Embase and Cochrane Library.

**Results:**

The initial search yielded original 440 articles. 15 studies were included in the final analysis, with 10 studies using bone removal software and 5 studies using virtual monochromatic imaging (VMI+). Pooled sensitivity and specificity for bone removal software for detecting significant stenosis below the knee was 94.8 % (95 % CI 88.1–97.8 %) and 59.3 % (95 % CI 43.3–73.6 %), respectively. All studies on VMI+ reported an increase in signal-to-noise ratio and contrast-to-noise ratio as the energy level decreased. Low energy VMI+ images had consistently higher qualitative imaging scores compared to both high energy and 120 kV blended reconstructions.

**Conclusion:**

DECTA provides high sensitivity and moderate specificity for detecting significant stenosis below the knee using bone removal software. Based on available literature, optimal imaging of BTK arteries can be achieved by using low energy VMI+ reconstructions.

## Highlights

• Dual energy bone removal software has high sensitivity and moderate specificity in lower leg arteries.
• Low energy dual energy images provide best qualitative imaging scores in lower leg arteries.
• Low energy reconstructions give high sensitivity and specificity, although evidence is limited.

## 1 Introduction

Dual energy computed tomography (DECT) is based on the acquisition of two x-ray beams with different energies. This allows for tissue characterisation, as tissues absorb and scatter radiation differently dependent on the x-ray energy [1]. In low energy beams, there will be an increase in the photoelectric effect, which is especially relevant in materials with high atomic numbers such as iodine. As the photon energy approaches the K-edge of iodine (33 keV), this results in a decreased tissue penetration and therefore increased tissue attenuation [2], [3]. However, this will increase image noise, which can be overcome by using noise-optimised post processing algorithms [4], [5].

In patients suffering from peripheral arterial disease (PAD), CT angiography (CTA) is widely used to identify the location and severity of atherosclerotic lesions, as well as to plan revascularisation procedures [6], [7]. In patients with both PAD and diabetes mellitus (DM), CTA can be particularly challenging, as DM is not only considered a leading cause for medial arterial calcifications [8], but also associated with atherosclerotic disease in BTK segments [9], [10]. On CTA, arterial wall calcifications can impede diagnostic accuracy for stenosis and occlusion due to blooming artefacts, especially in small arteries [11]. Additional challenges in lower leg imaging include reduced iodine signal due to upstream occlusions or stenosis, partial volume effects and outrunning the contrast bolus [12], [13]. These factors sometimes hinder correct grading of vascular stenosis, leading to negative digital subtraction angiographies (DSA).

Dual energy CTA can aid detection of luminal patency in atherosclerotic lesions by post processing algorithms including bone and plaque removal techniques and virtual monochromatic imaging (VMI). Bone and plaque removal software is based on material composition. It can differentiate and therefore subtract bone and calcified plaques from intravascular iodine. VMI simulates image acquisition at a chosen energy level that would result from image acquisition with a true mono-energetic X-ray beam [5]. With noise-optimised VMI reconstructions (VMI+), suboptimal contrast media in BTK segments can be enhanced to improve image quality using low energy reconstructions.

A number of studies and a systematic review [14] on image quality and diagnostic performance of DECTA of the pelvis and lower extremity have been published. However, few articles focus on image quality and diagnostic accuracy of below the knee (BTK) arteries specifically. To the best of our knowledge, there is no systematic review addressing DECTA of BTK arteries in patients suffering from PAD. Accordingly, the aim of this article is to conduct a systematic review of all published findings on the diagnostic performance of dual energy CTA of the lower legs in patients with PAD, including bone and plaque removal techniques and VMI.

## 2 Methods

### 2.1 Database search and study selection

The systematic review protocol followed the Preferred Reporting Items for Systematic Reviews and Meta-Analysis (PRISMA) 2020 statement guidelines [15]. Systematic searches of Pubmed, Embase and the Cochrane Library were performed in consultation with a research librarian on May 17th, 2024. The search strategy included terms related to dual energy computed tomography, peripheral artery disease and the lower extremity. The complete search strategy is available in appendix 1. After removal of duplicates, articles were manually screened for eligibility based on title and abstract by two independent reviewers. After this initial selection, full-text papers of eligible articles were obtained and screened independently by the same reviewers for final selection. Disagreement on eligibility was resolved by consensus. Reference lists of the included articles were checked to identify potentially missed relevant articles. This review was not registered and no protocol was prepared.

### 2.2 Inclusion criteria

Studies were eligible based on the following characteristics: 1) prospective and retrospective studies with DECTA series of BTK arteries in patients with PAD; 2) Assessment of imaging quality using subjective and/or objective parameters compared to blended images resembling 120 keV reconstructions; 3) or diagnostic accuracy (in terms of sensitivity and specificity) comparing DECTA to digital subtraction angiography (DSA) or conventional CTA. Only studies published in the English language were included. Phantom studies, case reports, review articles, letters to the editor and conference abstracts were excluded. No criteria on year of publication were applied.

### 2.3 Data extraction

One reader extracted all data, which was subsequently verified by another reader. Extracted data included publication details, patient characteristics, radiation dose and type of contrast medium. CT parameters included tube voltage, tube current, section thickness, reconstruction interval, gantry rotation time and pitch. Objective image quality based on image noise, signal-to-noise ratio (SNR) and contrast-to-noise ratio (CNR) was extracted. Subjective image quality based on different scoring scales was derived. Lastly, sensitivity and specificity were obtained to assess diagnostic accuracy. In this review, clinically significant stenosis was defined as ≥ 50 % lumen restriction. The full data extraction table is provided as appendix 2.

### 2.4 Statistical analysis and risk of bias

The methodological and statistical heterogeneity of the included studies did not allow for a meta-analysis. The I^2^ value for diagnostic accuracy in bone removal software was 39.1 %. The I^2^ value for SNR and CNR were 96.7 % and 99.4 %, respectively, indicating considerable heterogeneity. Therefore, we performed a narrative synthesis of the extracted data.

Primary outcomes were sensitivity and specificity for dual energy CTA compared to DSA as the reference standard. Statistical analyses were performed in R version 4.4.1 using RStudio 2024.09.0 (Rstudio, PBC, Boston). Sensitivity and specificity were pooled using the *mada* package, while CNR and SNR were pooled in the *meta* package. For all analyses, a random effects model was used.

Risk of bias was assessed using QUADAS-2 (Quality Assessment of Diagnostic Accuracy Studies) score [16]. Two reviewers independently asses the risk of bias for all included studies. Discrepancies were solved by consensus.

## 3 Results

**Fig. 1:**
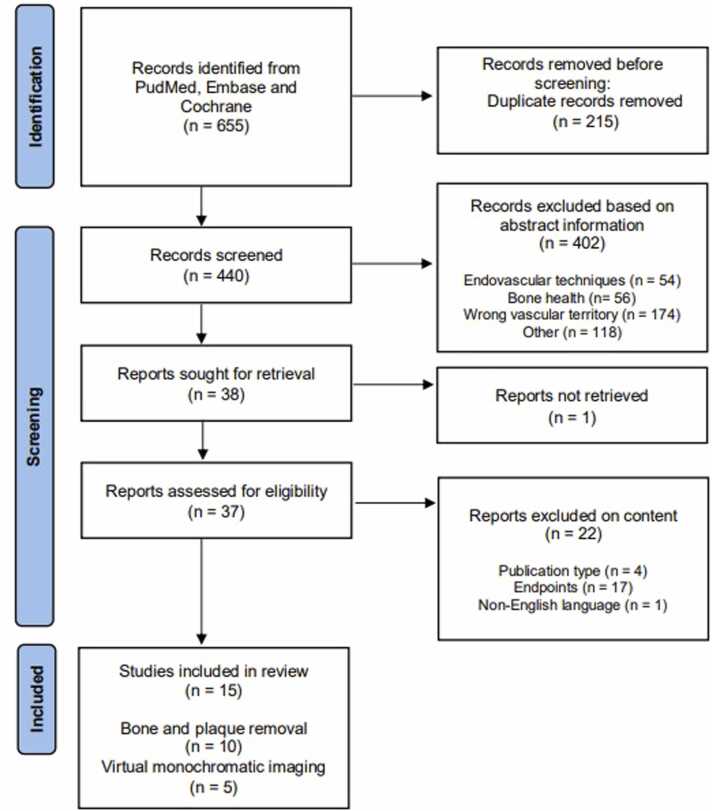
Study flowchart.

The initial search yielded 440 articles after removal of duplicates. A total of 440 articles were screened by reading the abstracts, and thirty-seven articles assessed for eligibility based on full text articles. Based on predefined inclusion criteria, 15 studies were included in the final analysis. Papers on low contrast volume (<70 ml) were excluded. Studies were categorised based on post processing techniques. Ten studies used bone and plaque removal software [17], [18], [19], [20], [21], [22], [23], [24], [25], [26], [27] and five studies focussed on VMI [28], [29], [30], [31], [32] (Fig. 1).

A total of 1060 patients were included in the final analysis, 443 in the VMI studies and 617 patients in the bone and plaque removal software studies. Patient characteristics and scan parameters are summarised in Table 1.

**Table 1:** Patient characteristics and scan parameters of all studies included in the final analysis. DSCT: dual source CT. N.A.: not available.

Study	No of patients	Mean age	Technique used for DECTA	Rotation time (s)	Pitch	Collimation	Section thickness (mm)	Increment (mm)	Tube voltage (tube A/B)	Tube current (tube A/B)	Contrast bolus (ml)	Flow rate (ml/s)
Brockmann et al., 2008	20	76	64 DSCT (2 tubes)	0,5	0.6	14 × 1.2	1.5	1	80/140	385/90	140 ml	4
Bucolo et al., 2023	77	79,2	384 DSCT (2 tubes)	0,5	0.6	2 × 192 x 0.6	1	1	90/150	73/45	N.A.	4
De Santis et al., 2019	88	65,9	384 DSCT (2 tubes)	N.A.	0.7	2 × 64 x 0.6	1.5	1	90/150	95/59	0.9 ml/kg, maximum 90 ml	3
Gruschwitz et al., 2022	118	75,6	384 DSCT (2 tubes)	0,25	0.3	128 × 0.6	1.5	1	90/150	150/94	110 ml	3
Huang et al., 2012	25	68	64 DSCT (2 tubes)	0,5	0,55	14 × 1.2	1.5	1	140/80	115/448	100 ml	3
Jia et al., 2020	182	66	256 slices CT (kilovolt switching)	1	0.98	N.A.	1.25	N.A.	140/80	185	95 ml	4 (first 55 ml), 2.5 (last 40 ml)
Kau et al., 2011	58	72,5	64 DSCT (2 tubes)	0,5	0,6	14 × 1.2	1.5	1	140/80	90/390	1,5 ml/kg Mean: 118 ml	4
Klink et al., 2017	94	72,7	64 DSCT (2 tubes)	0,5	1	64 × 1.2	1.5	1	140/80	50/270	80 ml	4
Koo et al., 2022	100	70,4	384 DSCT (2 tubes)	0,5	0.45	N.A.	1.5	1	150/100	69/124	1,5 ml/kg Mean: 93 ml	5 (first 30 ml), 3.5 (remaining bolus)
Kosmala et al., 2020	111	75	384 DSCT (2 tubes)	0,25	0.3	128 × 0.6	1.5	N.A.	90/150	150/94	110 ml	3
Meyer et al., 2008	50	67,7	64 DSCT (2 tubes)	0,33	0,65	2 × 32 x 0,6	1	0,7	140/80	56/238	100 ml	4
Sommer et al., 2009	51	70	64 DSCT (2 tubes)	0,5	0,7	64 × 0.6	1.5	1	140/80	80/340	160 ml	5,5
Sudarski et al., 2013	18	71	64 DSCT (2 tubes)	0,5	0,6	14 × 1.2	1.5	1	140/80	80/440	120 ml	5
Wichmann et al., 2016	48	63,3	384 DSCT (2 tubes)	N.A.	0.7	2 × 64 x 0.6	1.5	1	90 / 150	59/95	75 ml	2,5
Yamamoto et al., 2015	20	73	64 DSCT (2 tubes)	0,5	Adjusted per patient	2 × 32 x 0.6	1	0,75	140/80	95/405	120 ml	3,5

### 3.1 Radiation dose

Radiation dose was reported in eight studies [17], [18], [25], [26], [27], [28], [29], [31]. Average CTDIvol was 6.1 mGy for studies with a 64 slice DECT and 5.0 mGy for studies with a third generation 384 slice DECT. Average dose length product (DLP) was 917 mGycm for studies with a 64 slice DECT and 503.4 mGycm for studies with a third generation 384 slice DECT. Only one study [28] compared conventional CTA with DECTA, reporting a significant dose reduction (12 %) for DECTA.

### 3.2 Risk of bias

Concerns about risk of bias were noted in multiple studies in the following domains: patient selection (n = 4), index test (n = 6), reference standard (n = 9) and flow and timing (n = 5). Not performing DSA as gold standard resulted in low scores on the ‘reference standard’ domain.

Regarding applicability, concerns were raised regarding patient selection (n = 3) and reference standard (n = 3). Eventually, four studies were at low risk for bias and ten studies were judged as low concern regarding applicability. The overall quality of included studies on bone removal and VMI are presented in appendix 3.

### 3.3 Bone and plaque removal software

#### 3.3.1 Diagnostic accuracy

Seven studies reconstructed 3D maximum intensity projections (MIP) images with bone removal software, comparing these images to DSA [17], [22], [23], [24], [26] and other bone removal methods [19], [27] in BTK arteries. Three studies [22], [23], [25] assessed the grade of arterial stenosis visually, while one study [18] used an electronic caliper. Two studies [24], [26] graded stenosis visually, but used electronic calipers in unclear cases. Brockman et al. [17] did not specify how stenosis were graded.

**Fig. 2:**
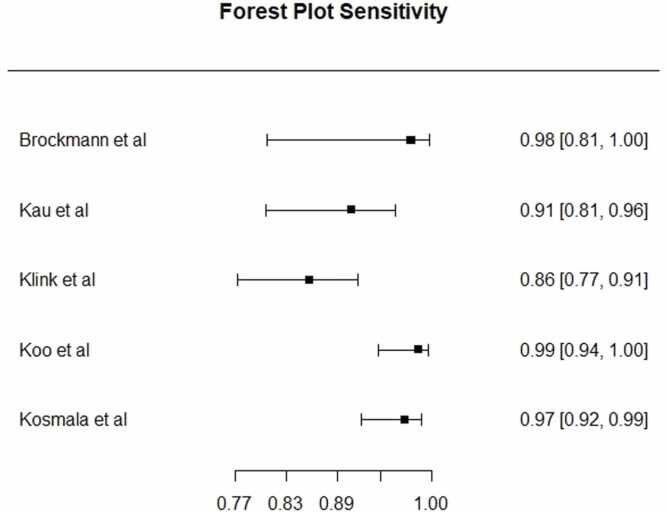
Forest plot for sensitivity for DECTA on 3D MIP in BTK arteries.

**Fig. 3:**
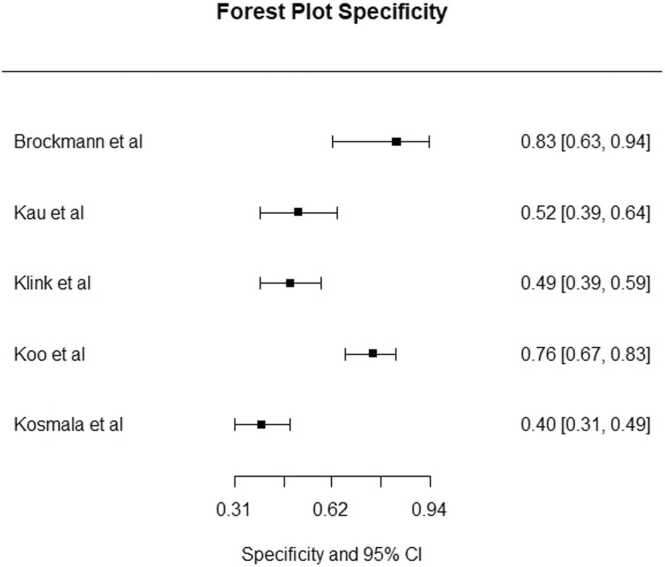
Forest plot for specificity for DECTA on 3D MIP in BTK arteries.

**Fig. 4:**
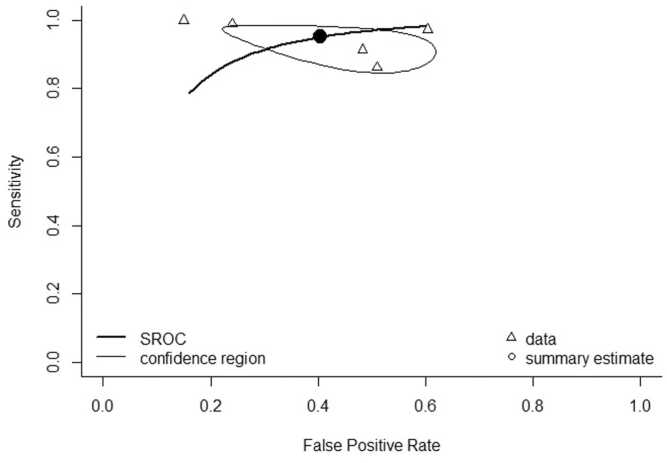
Sensitivity and false positive rates for DECTA on 3D MIP in BTK arteries, showing the pooled estimate (●) and individual studies (∆).

Two studies [18], [33] compared multiplanar reconstructions (MPR) images with bone removal to DSA and linear blend (Table 2). In studies comparing MPR bone removal software to DSA, pooled overall sensitivity and specificity was 96.4 % (95 % CI 93.1–98.2 %) and 81.8 % (95 % CI 74.0–87.6 %), respectively. Pooled sensitivity for DECTA for 3D MIP images compared to DSA in BTK arteries was in 94.8 % (95 % CI 88.1–97.8 %) (Fig. 2), and pooled specificity was 59.3 % (95 % CI 43.3–73.6 %) (Fig. 3). Fig. 4 shows pooled sensitivity and false positive rate, showing heterogeneous values across studies. Five studies [17], [22], [23], [24], [26] found lower specificity below the knee compared to the thigh and femoral arteries.

**Table 2:** Study characteristics of DECTA bone and plaque removal software. *no data on BTK arteries. ⱡ Statistical significant. MIP: maximum intensity projection. MPR: multiplanar reformation.

**Study**	**No of patients**	**Technique**	**Compared to**	**Qualitative image analysis**	**Sensitivity (%) on stenosis**	**Specificity (%) on stenosis**	**Accuracy (%)**
Brockmann et al. (2008)	20	− DE Bone removal on 3D MIP − Convetional bone removal	DSA	N.A.	DE bone remval: 100 %	DE bone remval: 86.9 %	DE bone remval: 90.9 %
					Conventional bone removal: 83.7	Conventional bone removal:53.4	Conventional bone removal: 62.4
De Santis et al. (2019)	88	− Calcium subtraction plaque removal, (MPR + 3D MIP) − Linear blend	DSA	Contrast opacification and calcium subtraction on 5 point scale	Plaque removal: 97.5*	Plaque removal: 95,6* ⱡ	Plaque removal: 96,5 * ⱡ
					Linear blend: 98.9*	Linear blend: 90.4* ⱡ	Linear blend: 93,1* ⱡ
Huang et al. (2012)	25	− Spectral iodine extraction − Linear blend	DSA	N.A.	N.A	N.A	N.A.
Kau et al. (2011)	53	Bone and plaque removal on 3D MIP	DSA	Quality of bone removal on 3 point scale	3D MIP: 91	3D MIP: 51	3D MIP: 74
Klink et al. (2017)	94	− Bone and plaque removal on (MPR + 3D MIP) − Linear blend	DSA	Overall image quality on 4 point scale	86	49	N.A.
Koo et al. (2022)	100	− Automatic plaque removal (APR) on axial MPR and 3D MIP − Linear blend	DSA	Overall image quality on 3 point scale	Linear blend: 98,11	Linear blend: 86,61	Linear blend: 92,36 ⱡ
					APR on 3D MIP: 99,06	APR on 3D MIP: 75,59	APR on 3D MIP: 87,32 ⱡ
					APR on linear blend: 98,11	APR on linear blend: 99,55	APR on linear blend: 94,33 ⱡ
Kosmala et al. (2020)	111	− DE bone and plaque removal on axial MPR and 3D MIP − Linear blend MPR and curved MPR − Automatic granding on linear blend	DSA	N.A.	Plaque removal 3D MIP: 97.6	Plaque removal 3D MIP: 40.0	Plaque removal 3D MIP: 91.5
					Plaque removal MPR: 98.8	Plaque removal MPR: 60.0	Plaque removal MPR: 94.7
					Linear blend MPR: 98.8	Linear blend MPR: 70.0	Linear blend MPR: 95.8
					Linear blend curved MPR: 97.6	Linear blend curved MPR: 87.5	Linear blend curved MPR: 96.7
					Automatic grading: 92.8	Automatic grading: 71.4	Automatic grading: 91.1
Meyer et al. (2008)	50	Automatic bone and plaque removal with manual bone removal	Linear blend, with manual bone subtraction	N.A.	Automatic bone and plaque removal with manual bone removal: 96.6	Automatic bone and plaque removal with manual bone removal: 38.7	N.S.
Sommer et al. (2009)	51	DE bone removal on 3D MIP	Software based bone removal on 3D MIP, with and without manual corrections	Overall image quality of bone removal on 3 point scale	N.A	N.A	N.A
Yamamoto et al. (2009)	20	DE bone removal axial MPR and 3D MIP	Manual bone removal	Vessel visibility and alterations on 3 point scale	N.A	N.A	N.A

Two studies comparing both DE and conventional bone removal to DSA [17], [24] demonstrated superior diagnostic accuracy for DE bone removal, with the highest rates for DE bone removal on axial images. However, Kosmala et al. reported the highest specificity rates for linear blend images. De Santis et al. did not provide data on BTK arteries specifically, but reported significantly higher specificity and accuracy in DE calcium subtraction compared to linear blend images.

Meyer et al. showed high sensitivity (96.6 %) but low specificity (38.7 %) for DE bone and plaque removal with manual bone removal using linear blend as golden standard. Three studies [19], [20], [27] did not provide data on diagnostic accuracy.

#### 3.3.2 Qualitative image analysis

Qualitative image analysis yielded heterogeneous results and was reported in six studies. In one study, overall image quality on DECTA was rated as ‘good’ or ‘excellent’ in 94.1 % [23]. Another study [24] found a percentage of 57.9 % of image quality rated as ‘good’ in BTK arteries on DECTA, compared to 95.2 % and 92.2 % in iliac and femoral arteries respectively.

De Santis et al. [18] found similar scores on visualisation of the arterial lumen in calcium subtraction images compared to standard linear blend.

Yamamoto et al. [19] reported significantly better vessel visibility below the knee with dual energy bone removal, compared to manual bone subtraction. One study [27] reported significantly fewer vessel segmentation errors with DE bone removal compared to software based bone removal. Another study [19] found no differences in vessel alterations between DE and manual bone subtraction. Image quality decreased in patients with calcified plaques [19], [27].

### 3.4 Virtual monochromatic imaging (VMI)

Five studies [29], [30], [31], [32], [33] addressed VMI at different energy levels compared to images constructed using standard linear blending, resembling single energy polychromatic 120 kV acquisitions. One study [32] did not use noise optimised VMI (VMI+), and one study did not specify the use of noise optimisation [28]. All studies showed increased CNR and SNR as energy levels decreased.

Pooled SNR for 50 and 55 keV VMI+ images BTK [28], [29], [30], [31] was 21.0 (95 % CI 16.7–25.3). Pooled CNR for 50 and 55 keV VMI+ images BTK [28], [29], [30], [31] was 25.44 (95 % CI 11.7–39.2). Three studies showed statistically significant higher SNR and CNR for low energy (40–50 keV) reconstructions compared to standard blended images [28], [29], [30]. Gruschwitz et al. [31] found no significant difference in SNR and CNR between low energy reconstructions and blended images, but low energy reconstructions were superior to 80–100 keV images. Sudarski et al. only reported superior CNR for 60 keV VMI acquisitions compared to stand blend. Study data is summarised in Table 3.

**Table 3:** Study characteristics of virtual monochromatic imaging (VMI). N.A.: not available. *no data on BTK arteries specifically. ⱡ Statistical significant.

**Study**	**No of patients**	**Different keV reconstructions**	**Compared to**	**VMI/VMI+**	**Qualitative image assessment**	**SNR (SD)**	**CNR (SD)**	**Sensitivity (%) on stenosis**	**Specificity (%) on stenosis**
Bucolo et al. (2023)	77	40, 55, 70, 85, 100	Linear blending ∼ 120 kV	VMI+	Image quality, image noise and vessel contrast on 5 point scale	55 keV: 23,28 (0,76)	55 keV: 21,46 (0,78)	N.A.	N.A.
Gruschwitz et al. (2022)	118	40, 55, 70, 85, 100	- Linear blending ∼ 120 kV - DSA < 30 days	VMI+	Intraluminal attenuation and image noise* (5 point scale)	50 keV: 25,4 (12,2)	50 keV: 21,6 (12,0)	50 keV: 100 %	50 keV: 100 %
								120 keV: 100 %	120 keV: 100 %
Jia et al. (2020)	182	50	- Conventional CTA at 100 kV - DSA < 180 days	N.A.	Overall Image quality on 4 point scale	50 keV: 19,92 (9,38)	50 keV: 45,60 (16,61)	50 keV: 92.8 ⱡ	50 keV: 97.34 ⱡ
								100 keV: 84.80	100 keV: 90.18
Sudarski et al. (2013)	18	40, 50, 60, 80, 90, 100, 110, 120	Linear blending ∼ 120 kV	VMI	N.A.	60 keV: 5 (3)	60 keV: 66 (43)	N.A.	N.A.
Wichmann et al. (2016)	48	40, 50, 60, 80, 90, 100, 110, 120	- Linear blending ∼ 120 kV - DSA < 30 days (n = 21)	VMI and VMI+	Intraluminal attenuation and image noise* (5 point scale)	VMI + 50 keV: 15,2 (7,0)	VMI+ 50 keV: 12,9 (6,7)	50 keV: 92 (CI 86–97) * ⱡ	50 keV: 95 (CI 92–98) * ⱡ
						VMI 50 keV: 8,8 (4,2)	VMI 50 keV: 7,6 (4,1)	120 keV: 87 (CI 78–96)*	120 keV: 90 (CI 85–95)*

#### 3.4.1 Diagnostic accuracy

Diagnostic accuracy was assessed in three studies, with one study [29] using an electronic caliper grade arterial stenosis, while the other two made subjective estimations [28], [31]. Jia et al. [28] found high sensitivity and specificity of 92.8 % and 97.3 % respectively for 50 keV images BTK arteries, with a significant difference compared to conventional CTA. Wichmann et al. [29] showed significantly higher overall sensitivity and specificity (92 % and 95 % respectively) for 50 keV VMI+ images compared to VMI and linear blend, but did not provide details on diagnostic accuracy below the knee. Gruschwitz [31] reported a 100 % sensitivity and specificity for occlusions of ≥ 75 % in both low energy reconstructions and blended images. However, they found 13 patients in which the lower leg runoff was only assessable in low energy VMI+ images.

#### 3.4.2 Qualitative image analysis

One study described qualitative image analysis for BTK segments specifically [28], reporting a significantly higher subjective image quality in 50 kV reconstructions for below the knee segments. Bucolo et al. [30] showed superior assessment on overall image quality, noise and diagnostic accessibility for 55 kV compared to high energy reconstructions and 120 kV blended images. Two studies [29], [31] using the same scale found significantly higher image quality scores for low energy reconstructions compared to high energy images and linear blend. Noise was significantly lower in VMI compared to VMI+ [29].

## 4 Discussion

This systematic review focusses on dual energy CTA with the use of bone and plaque removal software and VMI reconstructions in BTK arteries specifically. With regard to the use of bone removal software, our results indicate a high sensitivity of 94.8 % and a moderate specificity of 59.3 % for DECTA in BTK segments. Pooled sensitivity of the lower extremity, including iliac and femoral arteries, was comparable to sensitivity rates in the lower leg, suggesting that DECTA is a reliable tool to rule out PAD in BTK arteries. However, pooled specificity in BTK arteries was considerably lower than overall specificity, which implies overestimation of arterial stenosis in the lower legs. Our results are in line with a systematic review by Almutairi et al. [34], reporting a sensitivity and specificity of 95.5 % and 58.9 % respectively for BTK arteries and an overall sensitivity and specificity of 95.9 % and 79.8 %, respectively.

Arterial wall calcifications are common in BTK arteries in patients with PAD and diabetes mellitus and can lead to an overestimation of arterial stenosis [10], [35]. Multiple studies [23], [24], [26] found a significantly reduced specificity in calcified arteries, which might contribute to the moderate pooled specificity below the knee. Additionally, errors in bone and plaque removal can cause overestimation of stenosis [25], [36], especially below the knee, where the arteries are close to the tibia and fibula. As overestimation of arterial stenosis can lead to unnecessary invasive imaging and overtreatment, these results emphasise the need for further optimisation of DECTA protocols of the lower leg.

Post processing techniques using noise optimised VMI+ show high sensitivity (≥92 %) and specificity (≥ 97 %) for 50 keV VMI+ images in BTK arteries [28], [31]. Although limited studies have been published reporting on diagnostic accuracy in VMI+, these results are promising and the difference in specificity in VMI+ compared to bone removal software is noteworthy.

Results regarding qualitative image quality in DECTA with bone removal software were heterogeneous in both methods used for evaluation and results. Low energy VMI+ reconstructions had the highest qualitative image quality scores in all articles. However, only one article addressed subjective imaging data on BTK arteries specifically. Future research should include qualitative scores on the lower legs in both post processing techniques, because of the challenges this region imposes. Scoring systems should preferably address multiple domains including imaging quality, noise and vessel contrast, as described by Yuan et al. [37] and Bucolo et al. [30].

While CTA of the lower extremities is recommended as one of the primary imaging modalities in patients with PAD, current guidelines do not include recommendations on specific dual energy techniques [6], [7]. DECTA shows increased diagnostic accuracy in BTK arteries with the use of VMI+ dual energy techniques. Ideally, DECTA should be a helpful instrument to determine which patients will benefit from endovascular treatment, which patients can be treated conservatively and which patients require amputation due to poor circulation below the knee. This review is the first to include virtual monochromatic imaging and shows high sensitivity and specificity for low energy VMI+ reconstructions, indicating that this technique is suitable for evaluation of arterial runoff below the knee. All studies using noise optimized VMI+ found CNR and SNR rates higher than or equal to standard linear blended images for low energy reconstructions (40 – 55 keV) of the lower leg. However, all studies had a retrospective design and only one study compared conventional CTA to dual energy CTA [28]. Therefore, prospective, randomised studies on VMI+ DECTA of the lower leg are necessary to validate these results. To this end, the pedal arteries should also be included in this analysis, as there is currently no evidence on VMI+ in the foot arteries, while information on the pedal outflow is important for guiding decisions on endovascular treatment.

There are a few limitations to this review. First of all, the methodological and statistical heterogeneity of the included studies did not allow for a meta-analysis. Not all studies reported qualitative and objective image quality, diagnostic accuracy and dose reports, limiting our analysis. Only four studies had a prospective design and only one study directly compared conventional CTA and DECTA. Ten studies used DSA to calculate diagnostic accuracy, which is considered the ‘gold standard’ in PAD. An electronic calliper was used to objectively grade arterial stenosis in two studies. All other studies addressing diagnostic accuracy used subjective estimation to grade stenosis. Three studies reported the number of implanted vascular stents. Although vascular stents are less common in BTK arteries, no information on diagnostic implications regarding stents was provided. Additionally, the varied approaches to assess qualitative image quality impedes comparison across studies. Lastly, there was risk of bias in multiple included studies, due to study designs without DSA and missing information on blinding and scoring procedures.

In conclusion, dual energy CTA is an accurate diagnostic imaging technique to assess peripheral arterial disease below the knee, with high sensitivity. Bone removal software had moderate specificity with reduced image quality in BTK arteries compared to other vascular structures, presumably because of calcified plaques in these vessels. Low energy reconstructions with noise optimised VMI+ post processing provide optimal qualitative and objective image quality for assessment of BTK arteries. While VMI+ also show high diagnostic accuracy for stenosis and occlusion in BTK segments, evidence remains limited.

Before widespread implementation of low energy VMI+ reconstructions can be advocated, a prospective randomized controlled trial is needed, which should also take into account the diagnostic quality in the pedal arteries. Further optimisation of DECTA using VMI+ could be helpful to avoid over diagnosing of arterial stenosis and therefore help identify patients that will and will not benefit from endovascular treatment.

## CRediT authorship contribution statement

**Thijs Urlings:** Conceptualization, Writing – review & editing. **Van der Linden Edwin:** Writing – review & editing. **Panfilov Ilja:** Writing – review & editing. **Lodewijk Cobben:** Writing – review & editing. **De Jong Willem Jan:** Writing – review & editing. **Van der Vorst Joost:** Writing – review & editing. **Jaap Hamming:** Writing – review & editing. **Ayoub Charehbili:** Conceptualization, Data curation, Supervision, Writing – review & editing. **Jade Baars:** Data curation, Formal analysis, Writing – original draft.

## Informed Consent

Written informed consent was not required for this study because it concerns a review article.

## Declaration of Generative AI and AI-assisted technologies in the writing process

During the preparation of this work the author(s) did not use any AI tools.

## Compliance with Institutional Review Board and HIPAA Requirements

Institutional Review Board approval was not required because it concerns a review article. The results of this paper were not presented anywhere prior to publication.

## Funding statement

This study was funded by a grant from the Research Fund of Haaglanden Medical Center (2024).

## Declaration of Competing Interest

The authors declare that they have no known competing financial interests or personal relationships that could have appeared to influence the work reported in this paper.

## Footnotes

Appendix A Supplementary data associated with this article can be found in the online version at doi:10.1016/j.ejro.2025.100704.
