# Supplementary material for: The diagnostic value of dual-energy CTA for visualising below the knee arteries in peripheral arterial disease: A systematic review
Source: Eur J Radiol Open. 2025 Nov 7;15:100704. doi: 10.1016/j.ejro.2025.100704 (PMC12639553; doi:10.1016/j.ejro.2025.100704)
Supplement: Supplementary file 1 — Supplementary material [file mmc1.pdf]

Date	Database	Strategy	Number of references
17-05-2024	PubMed (www.pubmed.gov)	Combination 1:<br>("Chronic Limb-Threatening Ischemia"[Mesh] OR limb-threatening*[tiab] OR limbs-threatening*[tiab] OR limb-threating*[tiab] OR limbs-threating*[tiab] OR (limb*[ti] AND threat*[ti]) OR critical-limb*[tiab] OR limb-critical*[tiab] OR limbs-critical*[tiab] OR (limb*[ti] AND critical*[ti]) OR clti[tiab] OR critical-extremit*[tiab] OR extremities-critical*[tiab] OR extremity-critical*[tiab] OR (critical*[ti] AND extremit*[ti]) OR "Atherosclerosis"[Mesh] OR atherosclero*[tiab] OR athero-sclero*[tiab] OR aterosclero*[tiab] OR atero-sclero*[tiab] OR atherogen*[tiab] OR athero-gen*[tiab] OR aterogen*[tiab] OR atero-gen*[tiab] OR atheromat*[tiab] OR athero-mat*[tiab] OR ateromat*[tiab] OR atero-mat*[tiab] OR atheriosclero*[tiab] OR atherio-sclero*[tiab] OR ateriosclero*[tiab] OR aterio-sclero*[tiab] OR atheriogen*[tiab] OR atherio-gen*[tiab] OR ateriogen*[tiab] OR aterio-gen*[tiab] OR atheriomat*[tiab] OR atherio-mat*[tiab] OR ateriomat*[tiab] OR aterio-mat*[tiab] OR ((intralum*[tiab] OR intra-lum*[tiab]) AND plaque*[tiab]) OR peripheral-arter*[tiab] OR (peripher*[ti] AND arter*[ti]) OR paod[tiab]) AND ("Radiography, Dual-Energy Scanned Projection"[Mesh] OR dual-energ*[tiab] OR (dual*[ti] AND energ*[ti]) OR (dual*[tiab] AND (ct[tiab] OR cts[tiab] OR cta[tiab] OR computed-tomogra*[tiab] OR computed-x-ray-tomograph*[tiab] OR electron-beam-tomograph*[tiab] OR computerized-axial-tomograph*[tiab] OR computerized-tomograph*[tiab] OR computerised-axial-tomograph*[tiab] OR computerised-tomograph*[tiab] OR cat-scan*[tiab] OR computer-assisted-tomograph*[tiab] OR angiogra*[tiab] OR angio-gra*[tiab] OR angioradio*[tiab] OR angio-radio*[tiab] OR (blood*[ti] AND vessel*[ti] AND (radiograph*[ti] OR radio-graph*[ti])) OR ((blood-vessel*[tiab] OR bloodvessel*[tiab]) AND (radiograph*[tiab] OR radio-graph*[tiab])) OR vasculograph*[tiab] OR vasculo-graph*[tiab])) OR de-ct*[tiab]) AND ("Lower Extremity"[Mesh] OR extremit*[tiab] OR limb*[tiab] OR "leg"[tiab] OR legs*[tiab] OR crural*[tiab] OR foot*[tiab] OR feet*[tiab] OR popliteal*[tiab] OR femoropopliteal*[tiab] OR pedal*[tiab])	118.
		Combination 2:<br>("Chronic Limb-Threatening Ischemia"[Majr] OR (limb*[ti] AND threat*[ti]) OR (limb*[ti] AND critical*[ti]) OR clti[ti] OR (critical*[ti] AND extremit*[ti]) OR "Atherosclerosis"[Majr] OR atherosclero*[ti] OR athero-sclero*[ti] OR aterosclero*[ti] OR atero-sclero*[ti] OR atherogen*[ti] OR athero-gen*[ti] OR aterogen*[ti] OR atero-gen*[ti] OR atheromat*[ti] OR athero-	67.

		mat*[ti] OR ateromat*[ti] OR atero-mat*[ti] OR atheriosclero*[ti] OR atherio-sclero*[ti] OR ateriosclero*[ti] OR aterio-sclero*[ti] OR atheriogen*[ti] OR atherio-gen*[ti] OR ateriogen*[ti] OR aterio-gen*[ti] OR atheriomat*[ti] OR atherio-mat*[ti] OR ateriomat*[ti] OR aterio-mat*[ti] OR ((intralum*[ti] OR intra-lum*[ti]) AND plaque*[ti]) OR (peripher*[ti] AND arter*[ti]) OR paod[ti]) AND ("Radiography, Dual-Energy Scanned Projection"[Majr] OR (dual*[ti] AND (energ*[ti] OR ct[ti] OR cts[ti] OR cta[ti] OR computed-tomogra*[ti] OR computed-x-ray-tomograph*[ti] OR electron-beam-tomograph*[ti] OR computerized-axial-tomograph*[ti] OR computerized-tomograph*[ti] OR computerised-axial-tomograph*[ti] OR computerised-tomograph*[ti] OR cat-scan*[ti] OR computer-assisted-tomograph*[ti] OR angiogra*[ti] OR angio-gra*[ti] OR angioradio*[ti] OR angio-radio*[ti] OR (blood*[ti] AND vessel*[ti] AND (radiograph*[ti] OR radio-graph*[ti])) OR (bloodvessel*[ti] AND (radiograph*[ti] OR radio-graph*[ti])) OR vasculograph*[ti] OR vasculo-graph*[ti])) OR de-ct*[ti])	
17-05-2024	Embase - OVID-version	Search mode: Advanced.<br><br>Combination 1:<br>(exp critical limb ischemia/ OR (limb*.ti,ab,kf. ADJ3 threat*.ti,ab,kf.) OR (limb*.ti. AND threat*.ti.) OR (limb*.ti,ab,kf. ADJ3 critical*.ti,ab,kf.) OR (limb*.ti. AND critical*.ti.) OR clti.ti,ab,kf. OR (critical*.ti,ab,kf. ADJ3 extremit*.ti,ab,kf.) OR (critical*.ti. AND extremit*.ti.) OR exp atherosclerosis/ OR atherosclero*.ti,ab,kf. OR athero-sclero*.ti,ab,kf. OR aterosclero*.ti,ab,kf. OR atero-sclero*.ti,ab,kf. OR atherogen*.ti,ab,kf. OR athero-gen*.ti,ab,kf. OR aterogen*.ti,ab,kf. OR atero-gen*.ti,ab,kf. OR atheromat*.ti,ab,kf. OR athero-mat*.ti,ab,kf. OR ateromat*.ti,ab,kf. OR atero-mat*.ti,ab,kf. OR atheriosclero*.ti,ab,kf. OR atherio-sclero*.ti,ab,kf. OR ateriosclero*.ti,ab,kf. OR aterio-sclero*.ti,ab,kf. OR atheriogen*.ti,ab,kf. OR atherio-gen*.ti,ab,kf. OR ateriogen*.ti,ab,kf. OR aterio-gen*.ti,ab,kf. OR atheriomat*.ti,ab,kf. OR atherio-mat*.ti,ab,kf. OR ateriomat*.ti,ab,kf. OR aterio-mat*.ti,ab,kf. OR ((intralum*.ti,ab,kf. OR intra-lum*.ti,ab,kf.) AND plaque*.ti,ab,kf.) OR (peripher*.ti,ab,kf. ADJ3 arter*.ti,ab,kf.) OR (peripher*.ti. AND arter*.ti.) OR paod.ti,ab,kf.) AND (exp dual energy computed tomography/ OR (dual*.ti,ab,kf. ADJ3 energ*.ti,ab,kf.) OR (dual*.ti. AND energ*.ti.) OR (dual*.ti,ab,kf. AND (ct.ti,ab,kf. OR cts.ti,ab,kf. OR cta.ti,ab,kf. OR computed-tomogra*.ti,ab,kf. OR computed-x-ray-tomograph*.ti,ab,kf. OR electron-beam-tomograph*.ti,ab,kf. OR computerized-axial-tomograph*.ti,ab,kf. OR computerized-tomograph*.ti,ab,kf. OR computerised-axial-tomograph*.ti,ab,kf. OR computerised-tomograph*.ti,ab,kf. OR cat-scan*.ti,ab,kf. OR computer-assisted-tomograph*.ti,ab,kf. OR angiogra*.ti,ab,kf. OR angio-gra*.ti,ab,kf. OR angioradio*.ti,ab,kf. OR angio-radio*.ti,ab,kf.	301.

		OR (blood*.ti. AND vessel*.ti. AND (radiograph*.ti. OR radio-graph*.ti.)) OR ((blood-vessel*.ti,ab,kf. OR bloodvessel*.ti,ab,kf.) AND (radiograph*.ti,ab,kf. OR radio-graph*.ti,ab,kf.)) OR vasculograph*.ti,ab,kf. OR vasculo-graph*.ti,ab,kf.)) OR de-ct*.ti,ab,kf.) AND (exp lower limb/ OR extremit*.ti,ab,kf. OR limb*.ti,ab,kf. OR "leg".ti,ab,kf. OR legs*.ti,ab,kf. OR crural*.ti,ab,kf. OR foot*.ti,ab,kf. OR feet*.ti,ab,kf. OR popliteal*.ti,ab,kf. OR femoropopliteal*.ti,ab,kf. OR pedal*.ti,ab,kf.)<br><br>Combination 2:<br>(exp *critical limb ischemia/ OR (limb*.ti. AND threat*.ti.) OR (limb*.ti. AND critical*.ti.) OR clti.ti. OR (critical*.ti. AND extremit*.ti.) OR exp *atherosclerosis/ OR atheroscler o*.ti. OR athero-sclero*.ti. OR aterosclero*.ti. OR atero-sclero*.ti. OR atherogen*.ti. OR athero-gen*.ti. OR aterogen*.ti. OR atero-gen*.ti. OR atheromat*.ti. OR athero-mat*.ti. OR ateromat*.ti. OR atero-mat*.ti. OR atheriosclero*.ti. OR atherio-sclero*.ti. OR ateriosclero*.ti. OR aterio-sclero*.ti. OR atheriogen*.ti. OR atherio-gen*.ti. OR ateriogen*.ti. OR aterio-gen*.ti. OR atheriomat*.ti. OR atherio-mat*.ti. OR ateriomat*.ti. OR aterio-mat*.ti. OR ((intralum*.ti. OR intra-lum*.ti.) AND plaque*.ti.) OR (peripher*.ti. AND arter*.ti.) OR paod.ti.) AND (exp *dual energy computed tomography/OR (dual*.ti. AND (energ*.ti. OR ct.ti. OR cts.ti. OR cta.ti. OR computed-tomogra*.ti. OR computed-x-ray-tomograph*.ti. OR electron-beam-tomograph*.ti. OR computerized-axial-tomograph*.ti. OR computerized-tomograph*.ti. OR computerised-axial-tomograph*.ti. OR computerised-tomograph*.ti. OR cat-scan*.ti. OR computer-assisted-tomograph*.ti. OR angiogra*.ti. OR angio-gra*.ti. OR angioradio*.ti. OR angio-radio*.ti. OR (blood*.ti. AND vessel*.ti. AND (radiograph*.ti. OR radio-graph*.ti.)) OR (bloodvessel*.ti. AND (radiograph*.ti. OR radio-graph*.ti.)) OR vasculograph*.ti. OR vasculo-graph*.ti.)) OR de-ct*.ti.)	126.
17-05-2024	Cochrane Library (www.cochranelibrary.com)	Search mode: Advanced Search, Search Manager.<br><br>Search limits: 'Search word variations' is *de*activated.<br><br>These search strings are split into seperate components that are combined *afterwards*:<br><br>Combination 1:<br>(((limb* AND threat*) OR (limb* AND critical*) OR (critical* AND extremit*) OR (peripher* AND arter*)):ti) OR (((limb* NEAR threat*) OR (limb* NEAR critical*) OR clti OR (critical* NEAR extremit*) OR atherosclero* OR athero NEXT sclero* OR aterosclero* OR atero NEXT sclero* OR atherogen* OR athero NEXT gen* OR aterogen* OR atero NEXT gen* OR atheromat* OR athero NEXT mat* OR ateromat* OR atero NEXT mat* OR atheriosclero* OR atherio NEXT sclero* OR ateriosclero* OR	28.

		aterio NEXT sclero* OR atheriogen* OR atherio NEXT gen* OR ateriogen* OR aterio NEXT gen* OR atheriomat* OR atherio NEXT mat* OR ateriomat* OR aterio NEXT mat* OR ((intralum* OR intra NEXT lum*) AND plaque*) OR (peripher* NEAR arter*) OR paod):ti,ab,kw)<br>AND<br>((dual* AND energ*):ti) OR ((dual*):ti,ab,kw AND (blood* AND vessel* AND (radiograph* OR radio NEXT graph*)):ti) OR (((dual* NEAR energ*) OR (dual* AND (ct OR cts OR cta OR computed NEXT tomogra* OR computed NEXT x NEXT ray NEXT tomograph* OR electron NEXT beam NEXT tomograph* OR computerized NEXT axial NEXT tomograph* OR computerized NEXT tomograph* OR computerised NEXT axial NEXT tomograph* OR computerised NEXT tomograph* OR cat NEXT scan* OR computer NEXT assisted NEXT tomograph* OR angiogra* OR angio NEXT gra* OR angioradio* OR angio NEXT radio* OR ((blood NEXT vessel* OR bloodvessel*) AND (radiograph* OR radio NEXT graph*)) OR vasculograph* OR vasculo NEXT graph*)) OR de NEXT ct*):ti,ab,kw)<br>AND<br>(extremit* OR limb* OR "leg" OR legs* OR crural* OR foot* OR feet* OR popliteal* OR femoropopliteal* OR pedal*):ti,ab,kw<br><br><br>Combination 2:<br>((limb* AND threat*) OR (limb* AND critical*) OR clti OR (critical* AND extremit*) OR atheroscler* OR athero NEXT sclero* OR aterosclero* OR atero NEXT sclero* OR atherogen* OR athero NEXT gen* OR aterogen* OR atero NEXT gen* OR atheromat* OR athero NEXT mat* OR ateromat* OR atero NEXT mat* OR atheriosclero* OR atherio NEXT sclero* OR ateriosclero* OR aterio NEXT sclero* OR atheriogen* OR atherio NEXT gen* OR ateriogen* OR aterio NEXT gen* OR atheriomat* OR atherio NEXT mat* OR ateriomat* OR aterio NEXT mat* OR ((intralum* OR intra NEXT lum*) AND plaque*) OR (peripher* AND arter*) OR paod):ti<br>AND<br>((dual* AND (energ* OR ct OR cts OR cta OR computed NEXT tomogra* OR computed NEXT x NEXT ray NEXT tomograph* OR electron NEXT beam NEXT tomograph* OR computerized NEXT axial NEXT tomograph* OR computerized NEXT tomograph* OR computerised NEXT axial NEXT tomograph* OR computerised NEXT tomograph* OR cat NEXT scan* OR computer NEXT assisted NEXT tomograph* OR angiogra* OR angio NEXT gra* OR angioradio* OR angio NEXT radio* OR (blood* AND vessel* AND (radiograph* OR radio NEXT graph*)) OR (bloodvessel* AND (radiograph* OR radio NEXT graph*)) OR vasculograph* OR vasculo NEXT graph*)) OR de NEXT ct*):ti	3.
