# Supplementary material for: The diagnostic value of dual-energy CTA for visualising below the knee arteries in peripheral arterial disease: A systematic review
Source: Eur J Radiol Open. 2025 Nov 7;15:100704. doi: 10.1016/j.ejro.2025.100704 (PMC12639553; doi:10.1016/j.ejro.2025.100704)
Supplement: Supplementary file 3 — Supplementary material [file mmc3.docx]

**Tabular presentation for QUADAS-2 results**

**Bone and plaque removal:**

| **Study** | **RISK OF BIAS** | | | | **APPLICABILITY CONCERNS** | | |
| --- | --- | --- | --- | --- | --- | --- | --- |
|  | **PATIENT SELECTION** | **INDEX TEST** | **REFERENCE STANDARD** | **FLOW AND TIMING** | **PATIENT SELECTION** | **INDEX TEST** | **REFERENCE STANDARD** |
| Brockmann et al | ☺ | ☺ | ☺ | ☺ | ☺ | ☺ | ☺ |
| De Santis et al | ☺ | ☺ | ☺ | ☹ | ☺ | ☺ | ☺ |
| Huang et al | ☺ | ? | ☹ | ☹ | ☺ | ☺ | ☺ |
| Kau et al | ☺ | ☺ | ☺ | ☺ | ☺ | ☺ | ☺ |
| Klink et al | ☺ | ☺ | ☺ | ☺ | ☺ | ☺ | ☺ |
| Koo et al | ☹ | ☺ | ☹ | ☹ | ☺ | ☺ | ☺ |
| Kosmala et al | ☺ | ☺ | ☺ | ☺ | ☺ | ☺ | ☺ |
| Meyer et al | ☺ | ☺ | ☹ | ☺ | ☹ | ☺ | ☺ |
| Sommer et al | ☺ | ☹ | ☹ | ☺ | ☺ | ☺ | ☹ |
| Yamamoto et al | ☺ | ? | ☹ | ☺ | ☺ | ☺ | ☹ |

☺Low Risk ☹High Risk ? Unclear Risk

**Virtual monochromatic imaging (VMI):**

| **Study** | **RISK OF BIAS** | | | | **APPLICABILITY CONCERNS** | | |
| --- | --- | --- | --- | --- | --- | --- | --- |
|  | **PATIENT SELECTION** | **INDEX TEST** | **REFERENCE STANDARD** | **FLOW AND TIMING** | **PATIENT SELECTION** | **INDEX TEST** | **REFERENCE STANDARD** |
| Bucolo et al | ☹ | ☺ | ☹ | ☺ | ☹ | ☺ | ☺ |
| Gruschwitz et al | ☹ | ☺ | ☺ | ☺ | ☺ | ☺ | ☺ |
| Jia et al | ☹ | ? | ? | ☹ | ☹ | ☺ | ☹ |
| Sudarski et al | ☺ | ? | ☹ | ☺ | ☺ | ☺ | ☺ |
| Wichmann et al | ☺ | ? | ? | ☹ | ☺ | ☺ | ☺ |

☺Low Risk ☹High Risk ? Unclear Risk

**Graphical presentation for QUADAS-2 results, sorted by risk of bias and applicability**
